# Influencing Factors and Machine Learning-Based Prediction of Side Effects in Psychotherapy

**DOI:** 10.3389/fpsyt.2020.537442

**Published:** 2020-12-03

**Authors:** Lijun Yao, Xudong Zhao, Zhiwei Xu, Yang Chen, Liang Liu, Qiang Feng, Fazhan Chen

**Affiliations:** ^1^Shanghai Pudong New Area Mental Health Center, Tongji University School of Medicine, Shanghai, China; ^2^Department of Psychosomatic, Shanghai East Hospital, Tongji University School of Medicine, Shanghai, China; ^3^School of Computer Science, Fudan University, Shanghai, China

**Keywords:** side effects, psychotherapy, machine learning, online survey, China

## Abstract

**Background:** Side effects in psychotherapy are a common phenomenon, but due to insufficient understanding of the relevant predictors of side effects in psychotherapy, many psychotherapists or clinicians fail to identify and manage these side effects. The purpose of this study was to predict whether clients or patients would experience side effects in psychotherapy by machine learning and to analyze the related influencing factors.

**Methods:** A self-compiled “Psychotherapy Side Effects Questionnaire (PSEQ)” was delivered online by a WeChat official account. Three hundred and seventy participants were included in the cross-sectional analysis. Psychotherapy outcomes were classified as participants with side effects and without side effects. A number of features were selected to distinguish participants with different psychotherapy outcomes. Six machine learning-based algorithms were then chosen and trained by our dataset to build outcome prediction classifiers.

**Results:** Our study showed that: (1) the most common side effects were negative emotions in psychotherapy, such as anxiety, tension, sadness, and anger, etc. (24.6%, 91/370); (2) the mental state of the psychotherapist, as perceived by the participant during psychotherapy, was the most relevant feature to predict whether clients would experience side effects in psychotherapy; (3) a Random Forest-based machine learning classifier offered the best prediction performance of the psychotherapy outcomes, with an F1-score of 0.797 and an AUC value of 0.804. These numbers indicate a high prediction performance, which allowed our approach to be used in practice.

**Conclusions:** Our Random Forest-based machine learning classifier could accurately predict the possible outcome of a client in psychotherapy. Our study sheds light on the influencing factors of the side effects of psychotherapy and could help psychotherapists better predict the outcomes of psychotherapy.

## Introduction

Psychotherapy is the process in which a trained professional therapist uses guided conversations to facilitate changes in thoughts, feelings, and behaviors (1). People receiving psychotherapy expect positive change because it has proven to be effective for most clients or patients (2). However, one issue that has not been seriously considered is that after an individual enters psychotherapy, symptoms or clinical outcomes may be aggravated or worsen, and even cause harm (3). Unfortunately, many psychotherapists or clinicians fail to identify and manage these side effects, mainly due to insufficient awareness of the side effects of psychotherapy (4–6). Most studies on the effects of psychotherapy to date have focused on positive outcomes, with little attention paid to negative effects. To better understand whether harmful outcomes of psychotherapy were routinely collected and reported, a study analyzed 132 randomized, controlled trials. The researchers found that only 21% of these trials monitored harm to patients, and only 3% of the trials described adverse events (7).

A national survey (National Audit of Psychological Therapies, NAPT) conducted in England and Wales showed that 5.2% of people reported the long-lasting negative effects of psychotherapy (8). In a study about the adverse effects of psychotherapy in depressed patients (*n* = 135), 38.5% of patients reported having at least one side effect (9). Another study reported that the incidence of side effects in psychotherapy was 21%, and the most frequent side effects were “negative wellbeing/distress” (27% of patients), “worsening of symptoms” (9%), and “strains in family relations” (6%) (10). In outpatient cognitive behavioral therapy (CBT), up to 84% of outpatients reported having at least one unwanted side effect (11). It was estimated that the incidence of the adverse effects of psychotherapy, including long-lasting effects, was between 3 and 15% (12). Therefore, reports of the negative side effects of psychotherapy differed.

Many factors may affect the occurrence of side effects in psychotherapy. In the NAPT (8), people over 65 reported relatively few lasting negative effects of psychotherapy, while sexual and ethnic minorities were more likely to report them. Interestingly, when patients' treatment preferences were satisfied, they were more likely to report that the treatment had helped them solve their problems (13). Otherwise, they would experience more negative effects. The treatment preferences included “choice of venue,” “time of day of appointments,” “gender of the therapist,” “language/ interpreter,” and “type of treatment.” Therapist factors were also closely related to the outcomes of psychotherapy. The National Institute of Mental Health Treatment of Depression Collaborative Research Program (14) indicated that approximately 8% of the outcome variance in psychotherapy was attributed to the therapist. Another study showed that ~8% of the total variance and ~17% of the variance in rates of patient improvement could be attributed to the therapists (15). The personal attributes of the therapist, such as rigidity, uncertainty, criticism, alienation, tension, and distraction could negatively affect the outcomes of psychotherapy (16). In addition, many surveys have shown that the type of psychotherapy was also an important factor that affects side effects (8, 13, 17). Significantly more patients were treated with psychodynamic therapy and reported having “lasting negative effects” than those without psychodynamic therapy (8). Among the high-risk patients with side effects of psychotherapy, 11.6% were treated with CBT, 4.2% were treated with systemic therapy, 16.8% were treated with humanistic psychotherapy, and 67.2% were treated with psychodynamic therapy (17). In short, many factors are related to the side effects of psychotherapy, but we are still not sure which factors are the most relevant predictors of side effects in psychotherapy. Psychotherapists or clinicians cannot obtain a clear clinical practice outline of psychotherapy from past studies to reduce or avoid these side effects. Moreover, sensitivity to the side effects of psychotherapy is a characteristic of good therapists, which can significantly improve the quality of treatments (18). To solve these problems, our study implemented machine learning in the prediction of the side effects of psychotherapy.

Machine learning is a subfield of artificial intelligence, which builds a model to make a prediction or decision by learning from data. In the field of clinical psychology and psychiatry, this technique has been used for disease diagnosis, treatment prediction, and to some extent, the detection as well as the monitoring of potential biomarkers (19). There is currently no computational model that can predict whether a client/patient will experience side effects in different conditions. This study focuses on the side effects of psychotherapy, examining whether we can use machine learning technology to find out the potential clients/patients who might experience side effects in psychotherapy. This may have practical significance for improving the effectiveness of psychotherapy.

In the present study, we adopted six supervised machine learning-based models to predict whether clients or patients would experience side effects in psychotherapy, and compared the efficacy of these models to achieve the best prediction classifier. We analyzed various factors related to the generation of side effects and explored which factors were more relevant to these side effects. This research aims to provide psychotherapists with valuable information about the side effects of psychotherapy, thereby improving the effectiveness of daily clinical practice.

## Method

### Psychotherapy Side Effects Questionnaire (PSEQ)

Based on previous research results (20, 21), the “*Psychotherapy Side Effects Questionnaire (PSEQ)*” was compiled. In the PSEQ, the side effects in psychotherapy were defined as unwanted events that clients perceived during psychotherapy, which were inconsistent with expected goals and had a negative impact on clients. The side effects of psychotherapy were judged according to the answers to the first question: “Have you experienced any side effects or harm during your psychotherapy?”. An answer “yes” was considered to have side effects, otherwise, there was no indication of side effects. Seven questions in the PSEQ were designed to assess these side effects from three dimensions: symptoms, relationships, and social function (**Table 2**). Three questions were designed to assess the presence of new symptoms, which included negative emotions (Does psychotherapy make you feel bad?), bad behaviors (Does psychotherapy make you behave badly?), and physical discomfort (Does psychotherapy make your physical health uncomfortable?). One question was used to assess the original problem (Does psychotherapy make your problem worse?). Two questions were used to assess negative changes in family relationships (Does psychotherapy make your family relationship tense?), and interpersonal relationships (Does psychotherapy make your personal relationships tense outside of your family?). The last question was used to assess negative changes in social function (Does psychotherapy make your job worse?).

In order to predict the outcomes of psychotherapy, we collected the following information from each participant in the PSEQ: participant demographics (gender, age, marriage status, kids), whether they had received psychotherapy in the last 3 months (yes/no), the form of psychotherapy (face to face, phone, video), cost per psychotherapy, the effects of psychotherapy (invalid, limited effect, some effect, good effect, very effective, problem solved completely), the main causes of side effects in psychotherapy (the characters of psychotherapy skills, improper use of psychotherapy skills, limited professional ability of psychotherapists, client's mental activity, psychotherapist's mental activity, or other unpredictable factors), assessment and diagnosis by psychiatrists, medicine or physical therapy by psychiatrists, the willingness to seek psychotherapy in the future, the theoretical orientation of psychotherapy (psychoanalysis or psychodynamic therapy, cognitive behavioral therapy, humanistic therapy, narrative therapy, or unclear), and the place where psychotherapy took place (hospital, school, commercial psychological counseling agency, commercial psychological counseling network platform, others). Table 1 lists detailed information on each feature. The prepared questionnaire was sent to ten examiners for content feedback, and then revised again based on this feedback to create the final version of the PSEQ. In this survey, the Cronbach's α of the PSEQ is 0.74, indicated an acceptable internal consistency. The sociodemographic information and characteristics of the psychotherapy the participants received were also investigated.

**Table 1:** Features of participants included in the dataset.

**Features**	**With side- effects (*n* = 115)**	**Without side-effects (*n* = 255)**	**Overall**	***P*-value**
Gender				0.643
Male	14 (12.2%)	49 (19.2%)	63 (17.0%)	
Female	101 (87.8%)	206 (80.8%)	307 (83.0%)	
Age				0.029^*^
≤ 29	41 (35.7%)	89 (34.9%)	130 (35.1%)	
30-49	71 (61.7%)	145 (56.9%)	216 (58.4%)	
≥50	3 (2.6%)	21 (8.2%)	24 (6.5%)	
Marriage status				0.274
Single	40 (34.8%)	63 (24.7%)	103 (27.8%)	
Single with partner	12 (10.4%)	28 (11.0%)	40 (10.8%)	
Married	56 (48.7%)	152 (59.6%)	208 (56.2%)	
Divorced, separated or widowed	7 (6.1%)	12 (4.7%)	19 (5.1%)	
Kids				0.313
Yes	51 (44.3%)	148 (58.0%)	199 (53.8%)	
No	64 (55.7%)	107 (42.0%)	171 (46.2%)	
Psychotherapy at least once within the past 3 months				0.771
Yes	81 (70.4%)	189 (74.1%)	270 (73.0%)	
No	34 (29.6%)	66 (25.9%)	100 (27.0%)	
The form of psychotherapy				0.208
Face to face	88 (76.5%)	216 (84.7%)	304 (82.2%)	
Phone	9 (7.8%)	19 (7.5%)	28 (7.6%)	
Video	18 (15.7%)	20 (7.8%)	38 (10.3%)	
Cost (China Yuan/Time)				0.869
<200	25 (21.7%)	77 (30.2%)	102 (27.6%)	
200~400	45 (39.1%)	54 (21.2%)	99 (26.8%)	
400~600	19 (16.5%)	50 (19.6%)	69 (18.6%)	
600~800	15 (13.0%)	38 (14.9%)	53 (14.3%)	
>800	11 (9.6%)	36 (14.1%)	47 (12.7%)	
Effects of psychotherapy				0.011^*^
Invalid	17 (14.8%)	7 (2.7%)	24 (6.5%)	
Limited effect	21 (18.3%)	32 (12.5%)	53 (14.3%)	
Some effect	41 (35.7%)	100 (39.2%)	141 (38.1%)	
Good effect	23 (20.0%)	68 (26.7%)	91 (24.6%)	
Very effective	13 (11.3%)	47 (18.4%)	60 (16.2%)	
Problem solved completely	0 (0.0%)	1 (0.4%)	1 (0.3%)	
The main causes of side-effect in psychotherapy				
The characters of psychotherapy skills	34 (29.6%)	65 (25.5%)	99 (26.8%)	0.483
Improper use of psychotherapy skills	44 (38.3%)	75 (29.4%)	119 (32.2%)	0.165
Limited professional ability of psychotherapists	81 (70.4%)	127 (49.8%)	208 (56.2%)	0.014^*^
Client's mental activity	45 (39.1%)	151 (59.2%)	196 (53.0%)	0.014^*^
Psychotherapist's mental activity	63 (54.8%)	76 (29.8%)	139 (37.6%)	<0.001^*^
Other unpredictable factors	43 (37.4%)	120 (47.1%)	(4.1%)	0.195
Assessment and diagnosis by psychiatrists				0.622
Yes	54 (47.0%)	102 (40.0%)	156 (42.2%)	
No	61 (53.0%)	153 (60.0%)	214 (57.8%)	
Medicine or physical therapy by psychiatrists				0.738
Yes	47 (40.9%)	92 (36.1%)	139 (37.6%)	
No	68 (59.1%)	163 (63.9%)	231 (62.4%)	
The willingness to seek psychotherapy in the future				0.040^*^
Yes	79 (68.7%)	211 (82.7%)	290 (78.4%)	
No	6 (5.2%)	11 (4.3%)	17 (4.6%)	
Not sure	30 (26.1%)	33 (12.9%)	63 (17.0%)	
The theoretical orientation of psychotherapy				0.002^*^
Psychoanalysis or psychodynamic therapy	53 (46.1%)	81 (31.8%)	134 (36.2%)	
Cognitive behavioral therapy	12 (10.4%)	20 (7.8%)	32 (8.6%)	
Humanistic therapy	6 (5.2%)	8 (3.1%)	14 (3.8%)	
Family or couple therapy	15 (13.0%)	59 (23.1%)	74 (20.0%)	
Narrative therapy	6 (5.2%)	26 (10.2%)	32 (8.6%)	
Unclear	23 (20.0%)	61 (23.9%)	84 (22.7%)	
The place for psychotherapy				0.048^*^
Hospitals	29 (25.2%)	82 (32.2%)	111 (30.0%)	
Schools	11 (9.6%)	26 (10.2%)	37 (10.0%)	
Commercial psychological counseling agency	40 (34.8%)	107 (42.0%)	147 (39.7%)	
Commercial psychological counseling network platform	17 (14.8%)	17 (6.7%)	34 (9.2%)	
Others	18 (15.7%)	23 (9.0%)	41 (11.1%)	

* *P < 0.05 was considered statistically significant*.

### Procedure

The questionnaire was edited and released through the WeChat platform on February 11, 2019. WeChat is the leading mobile social network in China, with over 1 billion users. Participants read and decided whether to fill out the questionnaire according to the inclusion criteria. The questionnaire could only be submitted after participants agreed and gave their informed consent. The questionnaire was anonymous. The mode of dissemination was mainly based on reposting and sharing among WeChat users. Participants were encouraged to forward the questionnaire to various professional WeChat discussion groups of which they were part. They filled out the questionnaire online using the mobile phone interface provided by WeChat. The completion time for each questionnaire was about 3 min. Each WeChat user could only fill in the questionnaire once. The information collected by the questionnaire was automatically generated into an excel form. Data collection stopped on March 17, 2019.

### Participants

Participants were enrolled through an online questionnaire on their WeChat official account from February 11 to March 17, 2019. The inclusion criteria were: (1) that they had received at least one session of psychotherapy in the last six months; (2) that they were aged between 18 and 70 years old; and (3) that they gave informed consent. The exclusion criteria included: (1) a serious mental disorder with a risk of suicide and injury; (2) an education level below primary school; and (3) if they did not consent to the public release of research data.

### Machine Learning-Based Analysis

**Figure 1:**
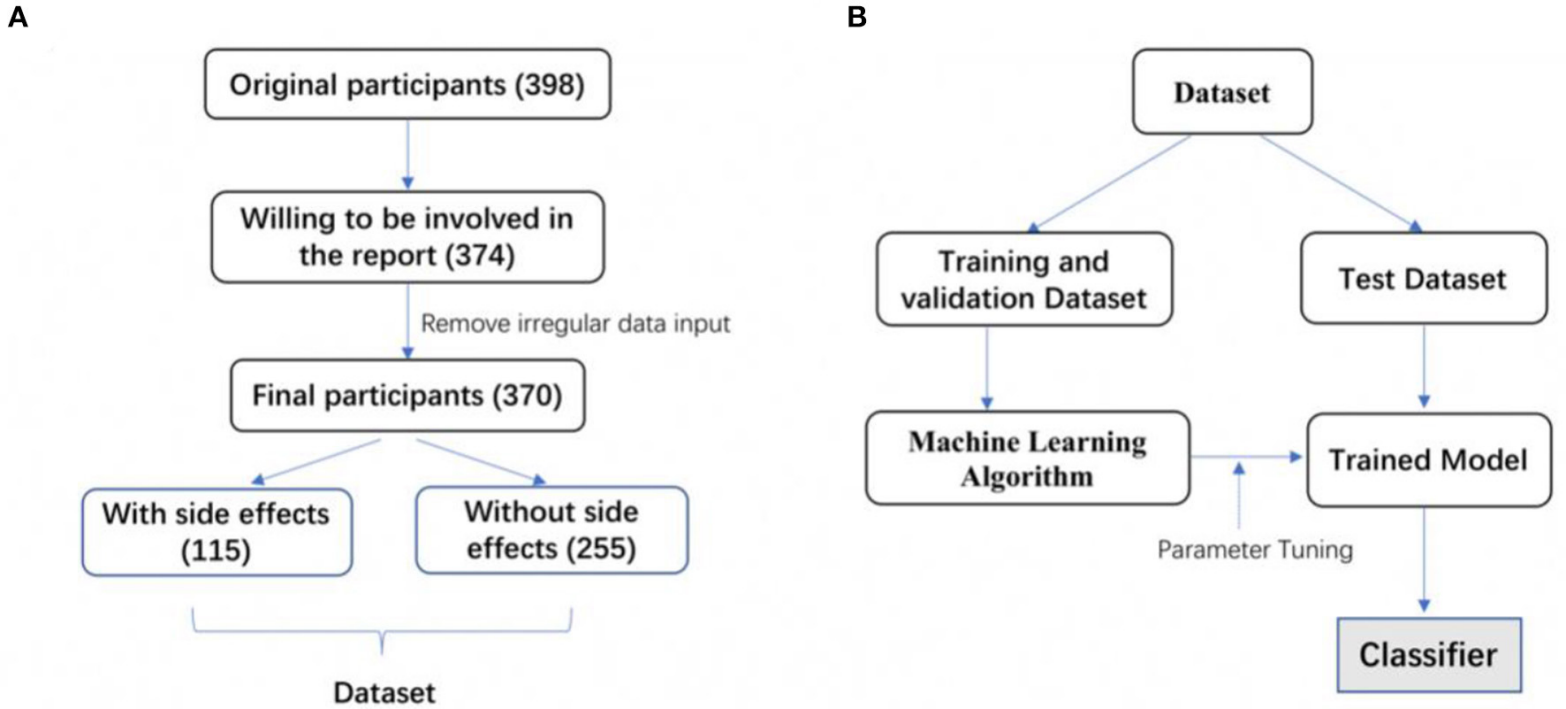
The workflow of data selection and machine-learning based modeling. **(A)** 398 participants were involved in the original questionnaire survey. By removing participants unwilling to make their data public and with irregular data input, 370 participants were finally involved in the dataset. One hundred and fifteen participants reported side effects, and 255 participants didn't report side effects. **(B)** The dataset was split into a training and validation dataset and a test dataset. Five different machine learning algorithms were selected for training based on the training and validation dataset. Trained models were obtained after parameter tuning. The final classifier was determined according to the comparison of each trained model's prediction performance.

We aimed to build a binary classifier that was able to distinguish participants with or without side effects from psychotherapy, based on their selection in the designed PSEQ. In our dataset, we chose participants “with side effects” category as the positive class. All the features used for machine learning analysis are described in Table 1. The process of our supervised machine learning-based analysis included the following steps: raw data preprocessing, feature selection, algorithm selection, parameter tuning, and performance evaluation. The workflow is described in Figure 1.

In the collected dataset, 115 participants reported having side effects from psychotherapy, while 255 participants had no or unclear side effects (“without side effects” group). To solve the unbalanced sample problem, we oversampled the minority type to 255 by the SMOTE technique (22). Then, we randomly split the entire dataset into a training and validation dataset and a test dataset. We used 70% of participants for training and validation and the remaining 30% for the test. We further used the 5-fold cross-validation method, where the training dataset was randomly divided into 5 subsets with equal sample sizes. Each of the 5 subsets was retained as validation data to evaluate the model, with the remaining 4 subsets used for training. The cross-validation process was repeated 5 times, with each of the 5 subsets used once for validation.

The machine learning algorithms selection used classical algorithms such as Random Forest (23), Logistic Regression (24), Support Vector Machine (SVM) (25), and AdaBoost (26), as well as emerging algorithms, i.e., XGBoost (27) and CatBoost (28). In particular, Random Forest is a widely used machine learning algorithm that uses a number of decision trees for learning. These decision trees collaborate as an ensemble to make the prediction. For a selected algorithm, we needed to determine an optimal set of parameters. Based on the training dataset, we applied a grid search to go through the parameter space. We selected a finite set of values for each parameter to form the parameter space. The grid search was iterated through a set of parameter combinations. For each combination, we evaluated prediction performance. Finally, we recorded the parameters leading to the maximum F1-score based on the training and validation dataset. Scikit-learn, a Python-based machine learning library, was used to train and evaluate the classification models (29).

For the model evaluation, we used precision, recall, F1-score, and the AUC (Area Under the ROC Curve) value to evaluate the prediction performance of our trained models (30). Specifically, precision is the fraction of participants with psychotherapy side effects classified by the model who did have side effects. The recall is the fraction of participants with side effects who had been correctly identified by the model. The F1-score is the harmonic mean of precision and recall, and was calculated as follows:

(1) $$\begin{array}{l} F1=\frac{2\times precision\times recall}{precision+recall} \end{array}$$

An F1-score reached its best value at 1 and the worst value at 0. From the perspective of psychologists, high precision means that our prediction rarely over reported and indicates that participants will likely have side effects when they are predicted with psychotherapy negative outcomes. Meanwhile, high recall means that our predictions rarely under report participants that will have side effects. A higher value of the F1-score indicates a better overall prediction performance of a classifier.

AUC is another important evaluation metric for examining the performance of a classification model and denotes the probability that a classifier will rank a random positive instance higher than a randomly chosen negative instance. The value of AUC is also between 0 and 1. For a perfect classifier, the AUC value will be 1. For a completely random classifier, the AUC value will be 0.5. In our work, the higher the AUC value, the better the model was at distinguishing participants with or without side effects from psychotherapy.

### Statistical Analysis

Statistical analyses used the Python programming language. The *P*-values in Table 1 were calculated by the Chi-Square test. *p* < 0.05 was considered statistically significant. We used the Chi-Square (χ^2^) statistics (31) to evaluate the dependence of a selected feature and the categories of participants (with or without side effects). We calculated the χ^2^ value based on the category information of participants and feature values. A larger χ^2^ value indicated a better discriminative power of a feature. According to the χ^2^ values, the top 8 ranked features that contributed most to differentiating participants with or without side effects from psychotherapy are presented in **Table 3**.

## Results

### Participant Demographics

A total of 398 participants filled in the PSEQ online. Twenty-eight participants (7.0%) were excluded from analysis because of their unwillingness to be included in published data or irregular data input. As a result, 370 participants were included for further analysis. The mean age of the participants was 34.6 years (SD = 10.4 years). The database comprised 14 main features, where each feature was either numerical or categorical. The detailed number, percentage, and classification of participants with each feature were shown in Table 1.

### The Types of Side Effects Experienced by Participants

Except for positive outcomes, many participants experienced different kinds of side effects in psychotherapy. Among the 370 participants, 115 participants reported having experienced side effects in psychotherapy. The incidence of side effects in the survey was 31.1%. The most common side effect was that participants “feel bad in psychotherapy” (24.6%), while the response “psychotherapy makes your job worse” (8.1%) was less common. In our PSEQ, we listed 7 types of common psychotherapy side effects. The detailed types and the incidence of each side effect are described in Table 2.

**Table 2:** The types of consulting side effects experienced by participants.

**Content of side-effects in the survey**	***n* (%)**
Does psychotherapy make you feel bad?	91 (24.6%)
Does psychotherapy make you behave badly?	41 (11.2%)
Does psychotherapy make your physical health uncomfortable?	40 (10.8%)
Does psychotherapy make your family relationship tense?	36 (9.7%)
Does psychotherapy make your personal relationship tense outside of your family?	33 (8.9%)
Does psychotherapy make your problem worse?	32 (8.6%)
Does psychotherapy make your job worse?	30 (8.1%)

### Feature Importance in Differentiating Participants With or Without Side Effects

The effectiveness of psychotherapy varied with the characteristics of each participant, as well as the different treatments provided by the psychotherapist. Next, we employed the Chi-Square statistics to quantify the discriminative power of each feature to the categories of participants. In total, 19 detailed features were included in this analysis. “Psychotherapist's mental activity” contributed most to the side effects of participants. The second highest ranked feature was “the theoretical orientation of psychotherapy.” The top 8 ranked features that have the greatest impact on distinguishing whether participants have side effects are listed in Table 3.

**Table 3:** The ranking of feature importance.

**Rank**	**Features**	**Chi-square value**
1	Psychotherapist's mental activity	13.163
2	The theoretical orientation of psychotherapy	9.715
3	Effects of psychotherapy	6.455
4	Client's mental activity	6.036
5	Limited professional ability of psychotherapist	6.001
6	Age	4.758
7	The willingness to seek psychotherapy in the future	4.228
8	The place for psychotherapy	3.906

**Figure 2:**
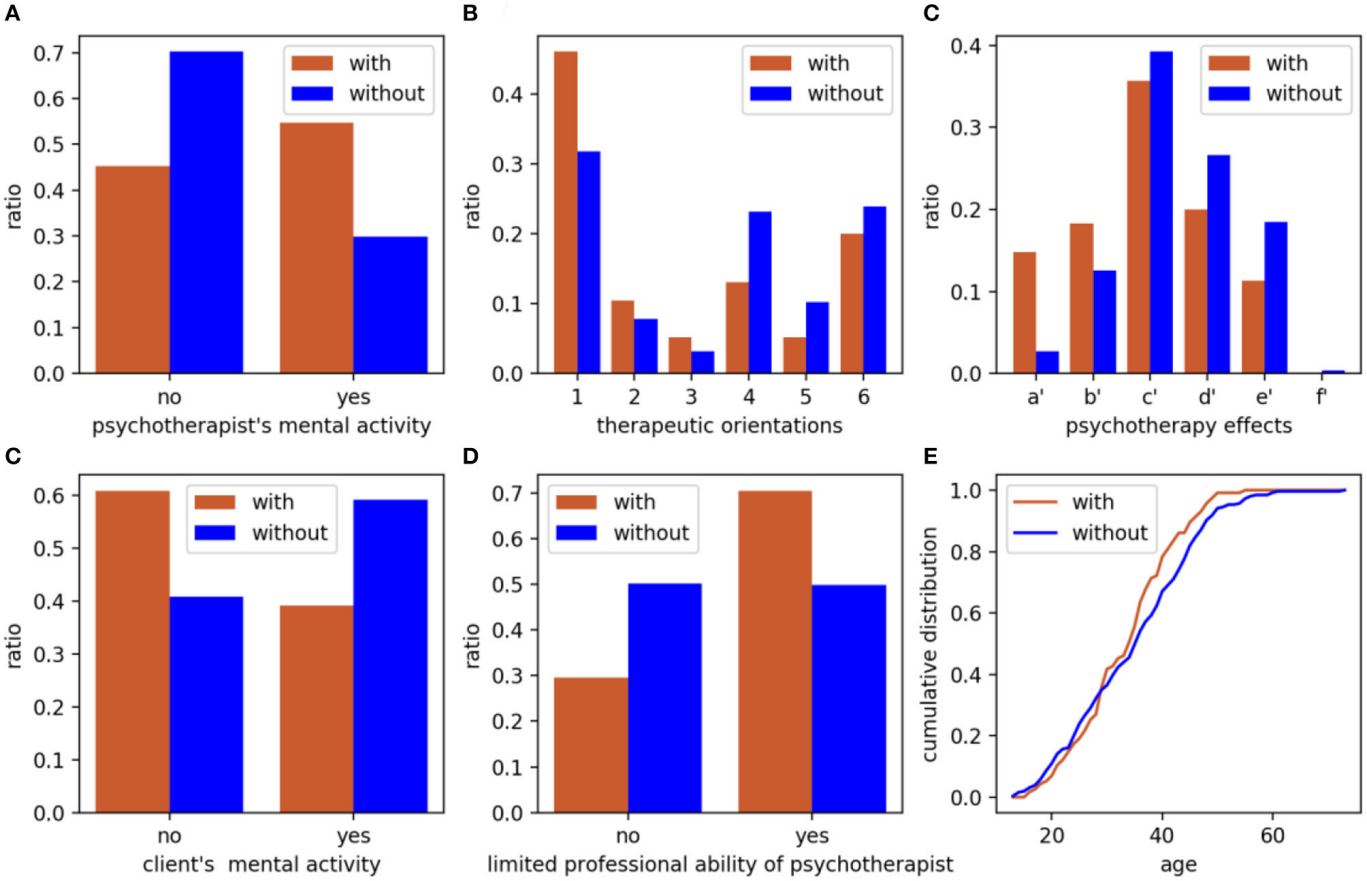
Comparison between participants with or without side effects based on graph metrics. **(A)** psychotherapist's mental activity; **(B)** therapeutic orientations, 1 to 6 denotes psychoanalysis or psychodynamic therapy, cognitive-behavioral therapy, humanistic therapy, family or couple therapy, narrative therapy and unclear, respectively; **(C)** effects of psychotherapy provided by participants; **(D)** client's mental activity, a′ to f′ denotes invalid, limited effect, some effect, good effect, very effective and problem solved completely, respectively; **(E)** limited professional ability of psychotherapist; **(F)** age. Brown column or line: participants with side effects; Blue column or line: participants without side effects.

To visualize the difference between participants with or without side effects, we compared the two groups of participants in terms of the psychotherapist's mental activity, the theoretical orientation of psychotherapy, the effects of psychotherapy, the client's mental activity, the limited professional ability of psychotherapist, and age, as shown in Figure 2. Participants who experienced side effects were more likely to think that the mental activity of the psychotherapist would cause harm to them, according to Figure 2A. Participants who experienced side effects were more concentrated in the middle age range, as shown in Figure 2F. Overall, we found that there were significant differences between the two groups in terms of the psychotherapist's mental activity, theoretical orientation, and the ability of psychotherapists, and the mental activity and age of clients.

### Machine Learning Algorithms and Predicting the Outcomes of Psychotherapy

In the present study, we employed supervised machine learning algorithms to predict whether a participant would experience side effects of psychotherapy treatment. In our dataset, 115 participants reported having side effects after psychotherapy, and 225 participants did not report side effects (Figure 1A). We then built a binary classifier that was able to classify participants with or without side effects more accurately. We used six different representative machine learning algorithms, Random Forest, XGBoost, CatBoost, Logistic Regression, SVM, and AdaBoost, to build classification models. Our results showed that the F1-scores of each of these six models (Random Forest, XGBoost, CatBoost, Logistic Regression, SVM, and AdaBoost) were 0.797, 0.788, 0.768, 0.765, 0.760, and 0.739, respectively (Table 4). Each model's precision and recall are also described in Table 4. The AUC values of each of these six models (Random Forest, XGBoost, CatBoost, Logistic Regression, SVM, and AdaBoost) were 0.804, 0.802, 0.772, 0.772, 0.765, and 0.735, respectively. Our data indicate that the Random Forest-based classifier achieved the highest F1-score of 0.797 and AUC value of 0.804, thus offering the best prediction between participants with or without side effects from psychotherapy.

**Table 4:** Comparison of the performance of different machine learning algorithms to predict the side effects in psychotherapy.

**Classifier**	**Precision**	**Recall**	**F1-Score**	**AUC**
Random Forest	0.787	0.808	0.797	0.804
XGBoost	0.812	0.767	0.788	0.802
CatBoost	0.744	0.795	0.768	0.772
Logistic Regression	0.750	0.781	0.765	0.772
SVM	0.740	0.781	0.760	0.765
AdaBoost	0.690	0.795	0.739	0.735

## Discussion

To the best of our knowledge, the present study was the first to explore the side effects of psychotherapy in a Chinese sample. This study analyzed the side effects of psychotherapy and the related factors that cause them and applied machine learning techniques to predict whether clients or patients would experience side effects. Based on our results, we concluded that: (1) the most common psychotherapy side effect was a negative emotion during psychotherapy, such as anxiety, tension, sadness, and anger, etc. (24.6%); (2) that the mental state of the psychotherapist, as perceived by the participant during psychotherapy, was most relevant in determining whether clients would experience side effects; and (3), that the Random Forest-based machine learning classifier offered the best prediction performance for distinguishing participants with or without side effects, with an F1-score of 0.797 and an AUC value of 0.804. In summary, our classifier can help therapists identify clients who may have side effects in psychotherapy, enabling therapists to provide patients/clients with better services.

In the survey, 31.1% of respondents reported experiencing side effects during psychotherapy. The most common side effect was that they “feel bad in psychotherapy” (24.6%). In the PSEQ, “feel bad” referred to a negative emotion experienced by participants in psychotherapy, such as anxiety, tension, sadness, and anger, etc. The results of our study were similar to those of previous studies (11, 12). However, more research has shown that the incidence of side effects in psychotherapy varied greatly from 3 to 84% (8, 11, 12, 20), and the clinical features were also different. The main reason for inconsistent results on the side effects of psychotherapy could be because there was no unified definition of side effects, and there was difficulty in selecting samples, especially concerning the influence that different theoretical approaches to psychotherapy can have on potential side effects (20, 21).

In the present study, we further analyzed the influencing factors related to psychotherapy side effects. Our results showed that the “psychotherapist's mental activity” was the most relevant feature in determining whether participants experienced side effects. In our survey, “psychotherapist's mental activity” referred to the psychotherapist's psychological state as deduced by the client during their interaction. Therapist factors mediate the outcomes of psychotherapy mainly through therapeutic alliance. On average, therapists who developed stronger alliances with their patients achieved better therapeutic results (32). According to Jennifer, Jonas, and Sylke (33), the negative effects of psychotherapy were particularly evident after a therapist had used controlling and challenging statements. In other words, failure to establish a strong therapeutic alliance between the therapist and the patient is a potential risk factor for treatment side effects. A good therapeutic alliance can be fostered in a supportive and reinforcing context, where less stressful interventions take place and the therapeutic relationship is comfortable. The therapist's activity and perceived mood affect patients through their therapeutic relationship, which was the most critical factor related to psychotherapy side effects in this study.

The “theoretical orientation” is the professional theoretical background of psychotherapy that the client learns from the therapist. Our results suggested that the theoretical application of psychotherapy had a significant predictive effect on the side effects experienced, which was consistent with previous studies (8, 13, 17). In our study, participants who received psychodynamic therapy had significantly higher rates of side effects than other treatments (Table 1). Leitner et al. (17) found that psychodynamic therapy had the highest risk of side effects in psychotherapy. Psychoanalysis or psychodynamic therapy focuses on the past life process based on defect orientation and externalizes internal conflicts into some traumatic events or experiences, which may cause the patient to attribute current difficulties to other people (especially parents), thus forming an isolated victim role (34). However, even though this therapeutic process is effective, it puts a lot of pressure on patients. Meanwhile, family therapy and other postmodernism psychotherapy (such as narrative therapy, solution-focused therapy) are more resource-oriented than system interactions, resources, and solutions, which may reduce the pressure on a client (8, 17).

Our study found also that other factors can cause side effects. These included the perceived limited professional abilities of the therapist, the client's mental activity, age, willingness to seek psychotherapy in the future, and the place where psychotherapy takes place. Parry, Crawford, and Duggan (35) conclude that the main factors that cause negative effects and harm in psychological therapies are as follows: (a) damaging interactions between the therapist and patient and unresolved ruptures in the therapeutic alliance; (b) therapist factors such as using an inappropriate therapeutic method or errors in delivering a recommended therapy; (c) patient factors that increase the risk of iatrogenesis; (d) a poor fit between therapist and patient; (e) the risks attached to specific interventions; and (f) organizational systems. Hardy et al. (12) have constructed a model of risk factors for negative experiences and describe how a “lack of fit” between patient needs, therapist skills, and service structures, could result in tensions between safety, containment, power, and control. This tension led to strain and poor engagement, resulting in a negative therapeutic experience. The side effects of psychotherapy involve a confluence of many factors, which should be considered a whole effect system between the therapist, the patient, and the organizational system.

Patients seek psychological treatment to solve problems and side effects do inevitably occur in some patients. Therefore, finding out which patients may have side effects is of great interest, and could provide useful information that will enable the therapist to obtain better results. In the present study, we demonstrated the usefulness of supervised machine learning algorithms in the prediction of side effects based upon information from participants as well as therapists. After evaluating a number of algorithms, we found that Random Forest-based classification is an effective tool to predict whether participants will experience side effects, with an F1-score of 0.797 and an AUC of 0.804. In the field of translational clinical psychology and psychiatry, machine learning has been widely used for disease diagnosis, differentiation, and outcome prediction (36, 37). In our study, we demonstrated that this classifier can accurately differentiate whether patients/clients are likely to experience side effects. For therapists, this result could have practical significance. If a client is predicted by the classifier as being potentially prone to side effects, the therapist could pay more attention to their treatment. Using the rank of feature importance, it is possible to adjust the treatment strategy. For example, the therapist could consider whether their mental state is stable, whether the therapy orientation adopted is suitable for the client, and so on, with the ultimate goal of better relieving or solving psychological problems. To the best of our knowledge, this is the first study to predict the potential side effects of psychotherapy using machine learning. The machine learning approaches described in this study are sufficiently accurate and meaningful and could be integrated into clinical psychology.

## Limitations

Although this study did develop an accurate model for predicting the side effects of psychotherapy, there are limitations connected to using PSEQ, a simple self-designed questionnaire, as the primary evaluation tool, meaning the validity and reliability of data on side effects might not be strong. At the same time, the participants conducted a self-assessment according to the inclusion criteria in the online survey which was disseminated via social media, which does not guarantee the validity or accuracy of the relatively small sample. That said, some important factors, such as treatment dosage and patient characteristics, were not included in the evaluation. This study did not explore which mental states or perceived moods of the therapist are likely to cause side effects in the client/patient, which could be the subject of future research.

## Conclusion

This study came to the following conclusions: (1), that the side effects experienced by patients during psychotherapy are common, and the most common side effect experienced by participants was negative emotion, such as anxiety, tension, sadness, and anger, etc.; (2), that the mental state of the therapist, as perceived by the participant during psychotherapy, was the most relevant feature in predicting whether clients would experience side effects; and (3), that our Random Forest-based machine learning model offered the best prediction performance of patient side effects after psychotherapy, with an F1-score of 0.797 and an AUC value of 0.804. In conclusion, these results could provide clinicians, therapists, and patients with important information that will help them to ensure that the side effects of psychotherapy are minimized or avoided in future clinical practice.

## Data Availability Statement

The datasets generated for this study are available on request to the corresponding author.

## Ethics Statement

The project involving human participants were reviewed and approved by the Ethics Committee of Shanghai Pudong New Area Mental Health Center, Tongji University School of Medicine (Approved Number: 2019tjdx9). The patients/participants provided their written informed consent to participate in this study.

## Author Contributions

LY and FC made substantial contributions to the conception, design, analysis, and manuscript draft, ensuring that the work was appropriately investigated and resolved. XZ, LL, and QF contributed to the study design and critical review of the manuscript for intellectual content. ZX and YC implemented machine learning algorithms and statistical analysis. All authors read and approved the final manuscript.

## Conflict of Interest

The authors declare that the research was conducted in the absence of any commercial or financial relationships that could be construed as a potential conflict of interest.
